# Supplementary material for: Structure determination of the HgcAB complex using metagenome sequence data: insights into microbial mercury methylation
Source: Commun Biol. 2020 Jun 19;3:320. doi: 10.1038/s42003-020-1047-5 (PMC7305189; doi:10.1038/s42003-020-1047-5)
Supplement: Supplementary file 1 — Supplementary Information [file 42003_2020_1047_MOESM1_ESM.pdf]

Supplementary Figures

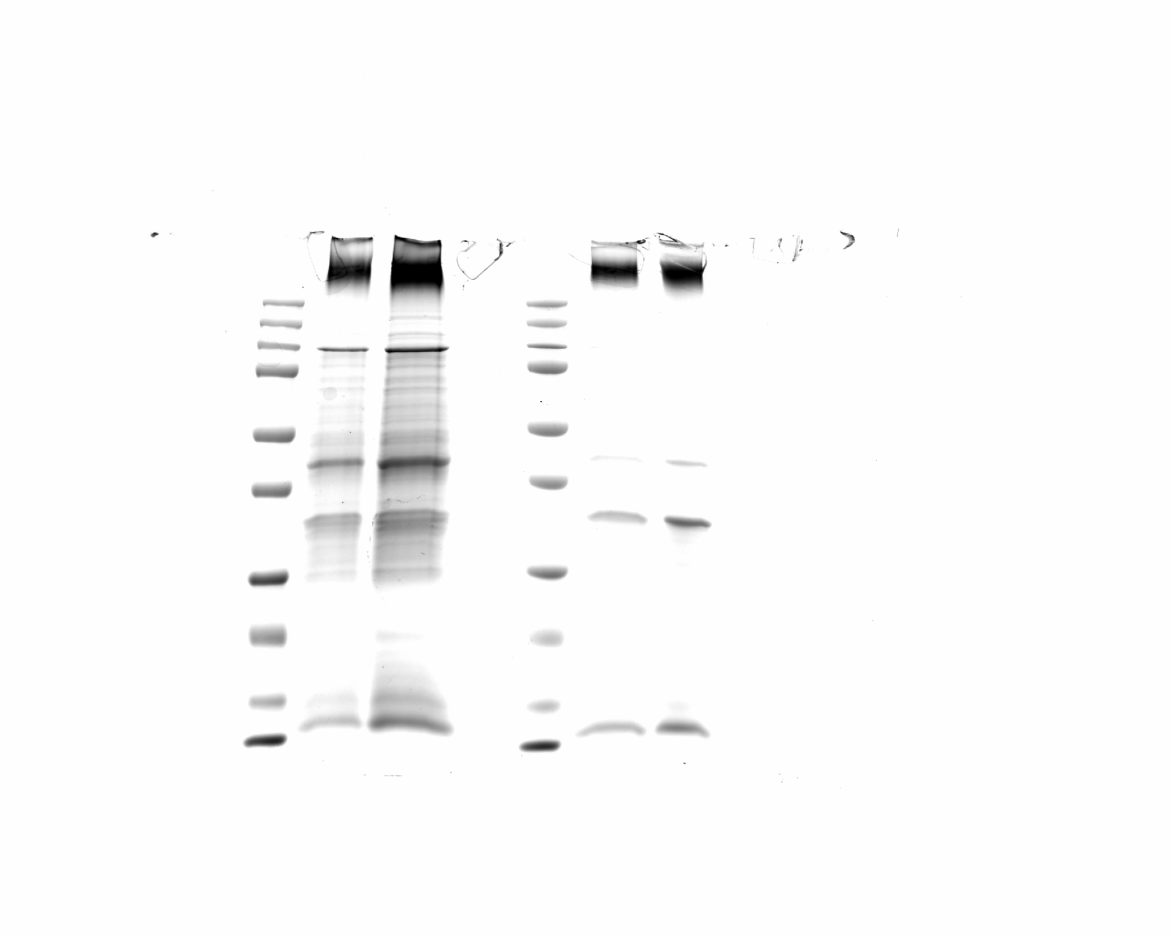

Supplementary Figure 1. Full, uncropped SDS-PAGE gel image of purified HgcA.

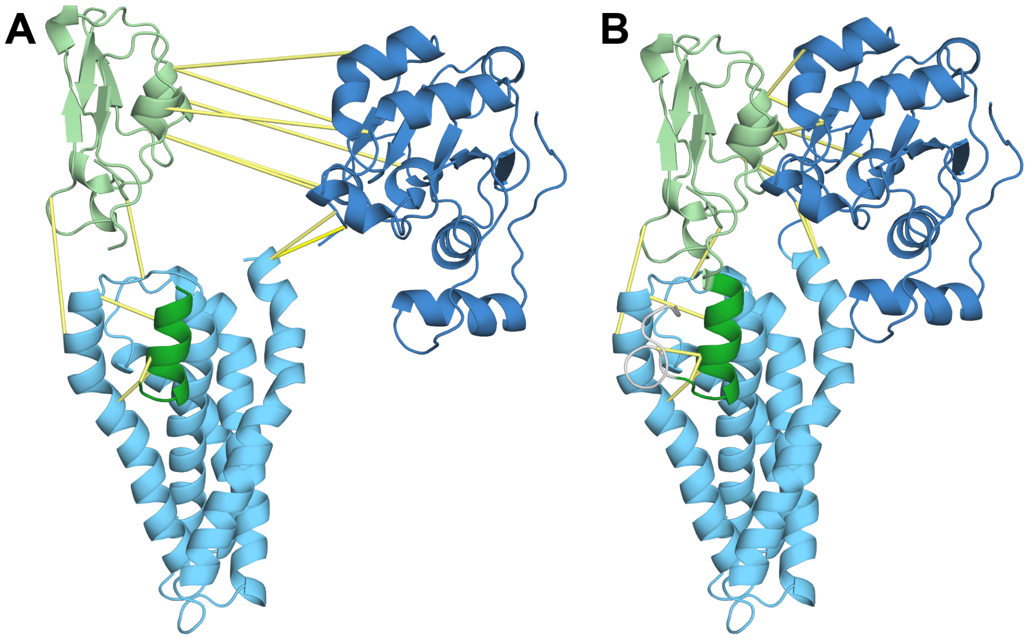

Supplementary Figure 2. (**A**) Top interdomain contacts in the HgcAB complex predicted from the coevolution analysis. (**B**) Interdomain contacts in the assembled HgcAB complex. *Colors*: Dark blue, CBD of HgcA; light blue, TMD of HgcA; light green, core of HgcB; dark green, C-terminal extension of HgcB. The most probable predicted interdomain contacts are shown in yellow.

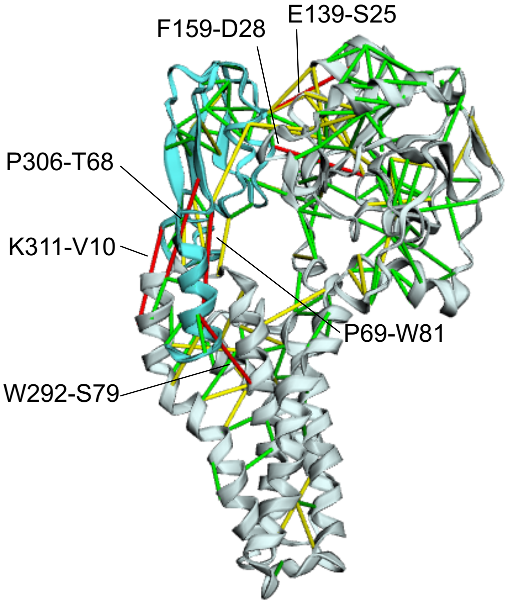

Supplementary Figure 3. Predicted contacts in the HgcAB model color coded by Cα-Cα distance. *Colors*: green (<5 Å), yellow (5-10 Å), red (>10 Å). Residues with distances >10 Å are labeled. All labels refer to the residue from HgcA followed by the residue from HgcB except P69-W81, for which both residues are from HgcB.

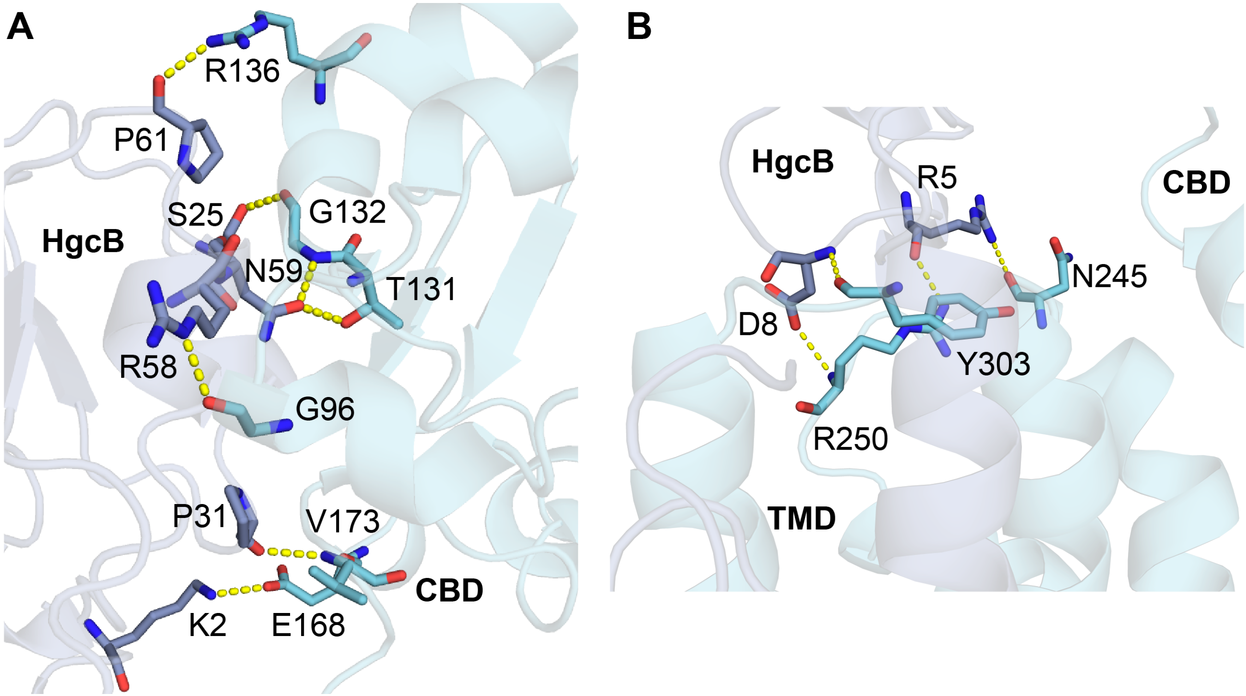

Supplementary Figure 4. Polar contacts between (**A**) CBD and HgcB and (**B**) TMD and HgcB.

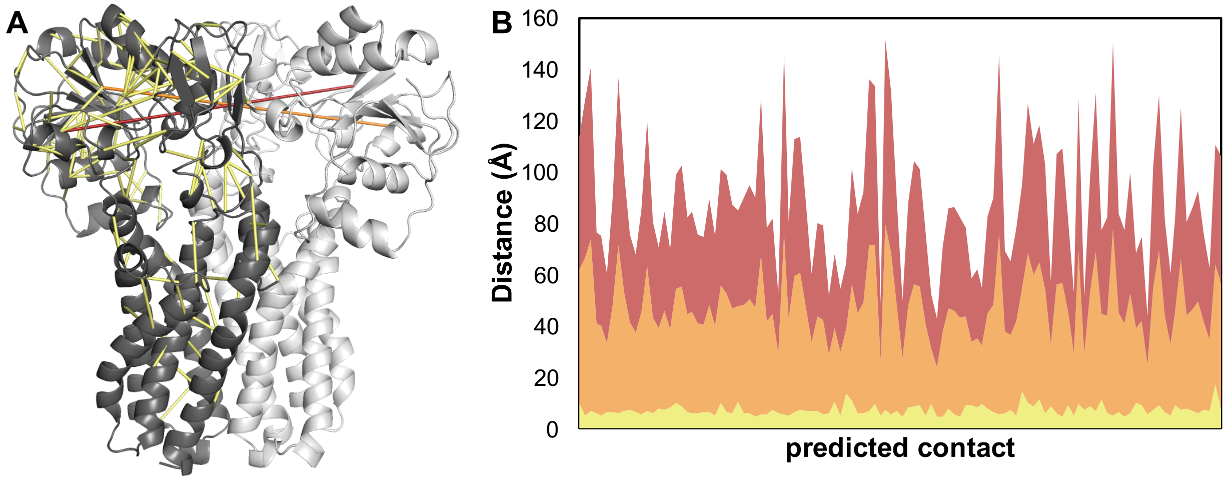

Supplementary Figure 5. (**A**) Dimer-of-heterodimers model generated by applying ambiguous restraints during symmetric docking of two HgcAB heterodimers. One HgcAB heterodimer is shown in dark gray and the other is shown in light gray. Predicted residue-residue contacts within a single HgcAB dimer are shown as yellow lines. Only contacts with probability >0.99 are shown. Representative examples of contacts that could potentially be satisfied between two separate heterodimers are shown as orange and red lines. (**B**) Stacked bar chart of possible contacts. The contacts are ordered by predicted probability with the highest on the left. In all cases the contacts within a single HgcAB heterodimer were shorter and therefore more favorable than inter-heterodimeric restraints, suggesting that the coevolution analysis supports a 1:1 HgcAB model rather than a 2:2 $(HgcAB)_2$ model.

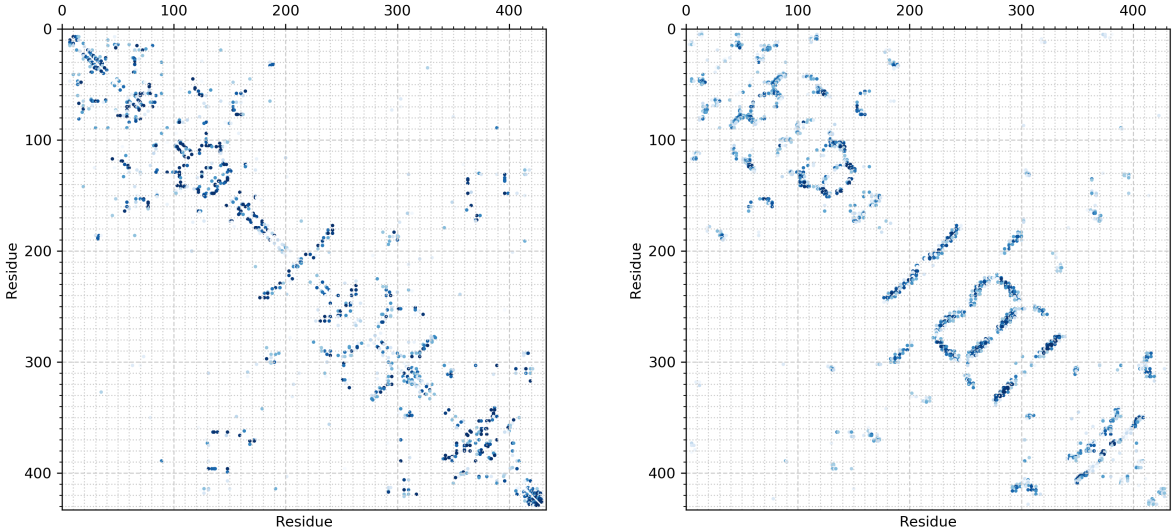

Supplementary Figure 6. Interresidue contact maps for HgcAB predicted by GREMLIN (*left*) and RaptorX-contact (*right*). A contact probability threshold of 0.5 was applied in both cases. Darker shades of blue indicate higher probability.

II. Supplementary Tables

Supplementary Table 1. HHsearch results for HgcAB

PDB ID	Coverage	Prob (%)	HHΔ	Description
4DJD_C	0.37	100	0.77	5-methyltetrahydrofolate corrinoid/iron sulfur protein methyltransferase
2H9A_A	0.37	100	0.78	CO dehydrogenase/acetyl-CoA synthase, iron-sulfur protein
1HFE_L	0.22	99.5	0.92	Fe-only hydrogenase
3GYX_B	0.19	99.4	0.93	adenylylsulfate reductase
1DWL_A	0.13	99.3	0.93	ferredoxin I
1F2G_A	0.13	99.3	0.93	ferredoxin II
1JNR_B	0.19	99.3	0.93	adenylylsulfate reductase
1XER_A	0.07	99.3	0.93	ferredoxin
4ID8_A	0.14	99.3	0.93	putative ferredoxin
1IQZ_A	0.16	99.3	0.93	ferredoxin

Supplementary Table 2. Top ten Dali results for the CBD of HgcA versus PDB25

PDB ID	Z-score	RMSD	LALI	N_res	%ID	Description
2YCL_A	14.2	2.7	135	442	27	carbon monoxide dehydrogenase corrinoid/iron-sulfur protein
3D0K_B	5.9	3.1	114	293	9	putative poly(3-hydroxybutyrate) depolymerase LpqC
2DST_A	5.5	3.5	101	122	16	hypothetical protein TTHA1544
3B48_F	5.1	3.2	98	135	5	uncharacterized protein
3GDW_B	4.9	3.6	101	138	5	sigma-54 interaction domain protein
2XDQ_A	4.8	3.8	96	425	7	light-independent protochlorophyllide reductase
3LFH_B	4.7	3.4	100	144	11	phosphotransferase system, mannose/fructose-specific component IIA
1CVR_A	4.6	3.1	98	433	7	gingipain R
6HSW_A	4.6	3.5	106	422	14	carbohydrate esterase family 15 domain protein
5ELM_B	4.4	3.5	99	236	16	Asp/Glu racemase family protein

Supplementary Table 3. Interactions between the B_{12} cofactor and residues in the CBD of HgcA

B_{12} atom	CBD atom	Distance (Å)
N3B	Thr60 (OG1)	2.9[a]
N3B	Ala61 (N)	3.3
O4	Thr66 (OG1)	3.0[a]
O2	Thr66 (OG1)	3.2
N52	Gly88 (O)	2.8
O51	Asn90 (N)	2.7
O3	Asn90 (ND2)	2.8
O4	Val91 (N)	3.0[a]
O5	Trp92 (N)	2.9
O39	Lys97 (NZ)	2.9
O7R	Gln127 (O)	3.0
O6R	Ala153 (N)	3.2[a]
O8R	Ala153 (N)	3.1

[a] Interaction present in CFeSP that was used as a distance restraint for docking B_{12} into the HgcAB model (see Methods for details).

Supplementary Table 4. Top ten Dali results for the TMD of HgcA versus PDB25

PDB ID	Z-score	RMSD	LALI	N_res	%ID	Description
2YVX_A	6.8	3.8	112	442	12	Mg^{2+} transporter MgtE
4TQ4_D	6.5	4.9	116	290	9	prenyltransferase
6IU4_A	5.6	3.7	84	225	10	iron transporter VIT1
5YCK_A	5.6	7.3	84	449	5	multidrug efflux transporter
6FV7_A	5.5	7.9	77	421	6	multidrug resistance transporter Aq_128
5EDL_A	5.4	4.5	115	197	6	S-component of ECF transporter, Putative HMP/thiamine permease protein YkoE
3FNB_A	5.4	4.9	102	374	6	acylaminoacyl peptidase SMU_737, hydrolase
5WEO_A	5.3	5.5	104	989	5	glutamate receptor 2, voltage-dependent calcium channel gamma-2 subunit chimera, transport protein
4IDN_B	5.3	4.7	88	423	3	atlastin-1, hydrolase
2XZE_A	5.1	2.9	86	141	7	stam-binding protein, hydrolase/transport

Supplementary Table 5. Top ten Dali results for HgcB versus PDB25

PDB ID	Z-score	RMSD	LALI	N_res	%ID	Description
5ODC_A	8.0	6.2	67	653	27	heterodisulfide reductase, subunit A
5T5M_F	7.9	3.4	67	342	31	tungsten formylmethanofuran dehydrogenase subunit FwdA
5T5I_P	7.8	1.3	57	81	39	tungsten formylmethanofuran dehydrogenase subunit FwdA
5OY0_c	7.8	2.9	64	81	27	photosystem I trimer
3GYX_B	7.7	4.3	61	166	33	adenylylsulfate reductase
5C4I_E	6.9	2.3	63	312	24	oxalate oxidoreductase subunit alpha
1SIZ_A	6.7	2.1	57	66	26	ferredoxin
3J16_B	6.4	6.9	72	608	24	ribosomal protein
1XER_A	6.2	1.4	57	103	35	ferredoxin
1IQZ_A	5.6	2.2	55	81	18	ferredoxin

Supplementary Table 6. Polar interactions between HgcA and HgcB in the HgcAB model

	HgcA	HgcB
CBD-HgcB (core)	Gly96 (O)	Arg58 (NE)
	Thr131 (OG1)	Asn59 (OD1)
	Gly132 (O)	Ser25 (OG)
	Gly132 (N)	Asn59 (OD1)
	Arg136 (NH1)	Pro61 (O)
	Glu168 (OE2)	Lys2 (NZ)
	Val173 (N)	Pro31 (O)
TMD-HgcB (tail)	Asn245 (O)	Arg5 (NH1)
	Arg250 (NH2)	Arg5 (O)
	Arg250 (N)	Asp8 (OD2)
	Tyr303 (O)	Asp8 (N)

Supplementary References

1. Rossmassler, K.; Dietrich, C.; Thompson, C.; Mikaelyan, A.; Nonoh, J. O.; Scheffrahn, R. H.; Sillam-Dusses, D.; Brune, A., Metagenomic analysis of the microbiota in the highly compartmented hindguts of six wood- or soil-feeding higher termites. *Microbiome* **2015,** *3*, 56.
2. Hervé, V.; Liu, P.; Dietrich, C.; Sillam-Dussès, D.; Stiblik, P.; Šobotník, J.; Brune, A., Phylogenomic analysis of 589 metagenome-assembled genomes encompassing all major prokaryotic lineages from the gut of higher termites. *PeerJ preprints* **2019**.
3. Lazar, C. S.; Baker, B. J.; Seitz, K. W.; Teske, A. P., Genomic reconstruction of multiple lineages of uncultured benthic archaea suggests distinct biogeochemical roles and ecological niches. *The ISME Journal* **2017,** *11* (5), 1118-1129.

4. Dombrowski, N.; Seitz, K. W.; Teske, A. P.; Baker, B. J., Genomic insights into potential interdependencies in microbial hydrocarbon and nutrient cycling in hydrothermal sediments. *Microbiome* **2017,** *5* (1), 106.
5. Angle, J. C.; Morin, T. H.; Solden, L. M.; Narrowe, A. B.; Smith, G. J.; Borton, M. A.; Rey-Sanchez, C.; Daly, R. A.; Mirfenderesgi, G.; Hoyt, D. W.; Riley, W. J.; Miller, C. S.; Bohrer, G.; Wrighton, K. C., Methanogenesis in oxygenated soils is a substantial fraction of wetland methane emissions. *Nat Commun* **2017,** *8* (1), 1567.
6. SeaBioTech http://spider.science.strath.ac.uk/seabiotech/
7. Graham, E. B.; Gabor, R. S.; Schooler, S.; McKnight, D. M.; Nemergut, D. R.; Knelman, J. E., Oligotrophic wetland sediments susceptible to shifts in microbiomes and mercury cycling with dissolved organic matter addition. *PeerJ* **2018,** *6*, e4575.
8. Fortney, N. W.; He, S.; Converse, B. J.; Beard, B. L.; Johnson, C. M.; Boyd, E. S.; Roden, E. E., Microbial Fe(III) oxide reduction potential in Chocolate Pots hot spring, Yellowstone National Park. *Geobiology* **2016,** *14* (3), 255-75.
9. Fortney, N. W.; He, S.; Kulkarni, A.; Friedrich, M. W.; Holz, C.; Boyd, E. S.; Roden, E. E., Stable Isotope Probing for Microbial Iron Reduction in Chocolate Pots Hot Spring, Yellowstone National Park. *Appl Environ Microbiol* **2018,** *84* (11).
10. Haynes, K. M.; Kane, E. S.; Potvin, L.; Lilleskov, E. A.; Kolka, R. K.; Mitchell, C. P. J., Mobility and transport of mercury and methylmercury in peat as a function of changes in water table regime and plant functional groups. *Global Biogeochemical Cycles* **2017**.
11. Haynes, K. M.; Kane, E. S.; Potvin, L.; Lilleskov, E. A.; Kolka, R. K.; Mitchell, C. P. J., Gaseous mercury fluxes in peatlands and the potential influence of climate change. *Atmospheric Environment* **2017,** *154*, 247-259.
12. Haynes, K. M.; Kane, E. S.; Potvin, L.; Lilleskov, E. A.; Kolka, R. K.; Mitchell, C. P. J., Impacts of experimental alteration of water table regime and vascular plant community composition on peat mercury profiles and methylmercury production. *Sci Total Environ* **2019,** *682*, 611-622.
13. Voorhies, A. A.; Eisenlord, S. D.; Marcus, D. N.; Duhaime, M. B.; Biddanda, B. A.; Cavalcoli, J. D.; Dick, G. J., Ecological and genetic interactions between cyanobacteria and viruses in a low-oxygen mat community inferred through metagenomics and metatranscriptomics. *Environ Microbiol* **2016,** *18* (2), 358-71.
14. Voorhies, A. A.; Biddanda, B. A.; Kendall, S. T.; Jain, S.; Marcus, D. N.; Nold, S. C.; Sheldon, N. D.; Dick, G. J., Cyanobacterial life at low O(2): community genomics and function reveal metabolic versatility and extremely low diversity in a Great Lakes sinkhole mat. *Geobiology* **2012,** *10* (3), 250-67.
15. Beller, H. R.; Rodrigues, A. V.; Zargar, K.; Wu, Y. W.; Saini, A. K.; Saville, R. M.; Pereira, J. H.; Adams, P. D.; Tringe, S. G.; Petzold, C. J.; Keasling, J. D., Discovery of enzymes for toluene synthesis from anoxic microbial communities. *Nat Chem Biol* **2018,** *14* (5), 451-457.
16. Zargar, K.; Saville, R.; Phelan, R. M.; Tringe, S. G.; Petzold, C. J.; Keasling, J. D.; Beller, H. R., In vitro characterization of phenylacetate decarboxylase, a novel enzyme catalyzing toluene biosynthesis in an anaerobic microbial community. *Sci Rep* **2016,** *6*, 31362.
17. Espinola, F.; Dionisi, H. M.; Borglin, S.; Brislawn, C. J.; Jansson, J. K.; Mac Cormack, W. P.; Carroll, J.; Sjoling, S.; Lozada, M., Metagenomic analysis of subtidal sediments from polar and subpolar coastal environments highlights the relevance of anaerobic hydrocarbon degradation processes. *Microb Ecol* **2018,** *75* (1), 123-139.

18. Tas, N.; Prestat, E.; Wang, S.; Wu, Y.; Ulrich, C.; Kneafsey, T.; Tringe, S. G.; Torn, M. S.; Hubbard, S. S.; Jansson, J. K., Landscape topography structures the soil microbiome in arctic polygonal tundra. *Nat Commun* **2018,** *9* (1), 777.
19. Kimbrel, J. A.; Ballor, N.; Wu, Y. W.; David, M. M.; Hazen, T. C.; Simmons, B. A.; Singer, S. W.; Jansson, J. K., Microbial community structure and functional potential along a hypersaline gradient. *Front Microbiol* **2018,** *9*, 1492.
20. Wrighton, K. C.; Thomas, B. C.; Sharon, I.; Miller, C. S.; Castelle, C. J.; VerBerkmoes, N. C.; Wilkins, M. J.; Hettich, R. L.; Lipton, M. S.; Williams, K. H.; Long, P. E.; Banfield, J. F., Fermentation, hydrogen, and sulfur metabolism in multiple uncultivated bacterial phyla. *Science* **2012,** *337* (6102), 1661-5.
21. Probst, A. J.; Ladd, B.; Jarett, J. K.; Geller-McGrath, D. E.; Sieber, C. M. K.; Emerson, J. B.; Anantharaman, K.; Thomas, B. C.; Malmstrom, R. R.; Stieglmeier, M.; Klingl, A.; Woyke, T.; Ryan, M. C.; Banfield, J. F., Differential depth distribution of microbial function and putative symbionts through sediment-hosted aquifers in the deep terrestrial subsurface. *Nat Microbiol* **2018,** *3* (3), 328-336.
22. Hug, L. A.; Thomas, B. C.; Brown, C. T.; Frischkorn, K. R.; Williams, K. H.; Tringe, S. G.; Banfield, J. F., Aquifer environment selects for microbial species cohorts in sediment and groundwater. *ISME J* **2015,** *9* (8), 1846-56.
23. Anantharaman, K.; Brown, C. T.; Hug, L. A.; Sharon, I.; Castelle, C. J.; Probst, A. J.; Thomas, B. C.; Singh, A.; Wilkins, M. J.; Karaoz, U.; Brodie, E. L.; Williams, K. H.; Hubbard, S. S.; Banfield, J. F., Thousands of microbial genomes shed light on interconnected biogeochemical processes in an aquifer system. *Nat Commun* **2016,** *7*, 13219.
24. Brown, C. T.; Hug, L. A.; Thomas, B. C.; Sharon, I.; Castelle, C. J.; Singh, A.; Wilkins, M. J.; Wrighton, K. C.; Williams, K. H.; Banfield, J. F., Unusual biology across a group comprising more than 15% of domain Bacteria. *Nature* **2015,** *523* (7559), 208-11.
25. Kantor, R. S.; Huddy, R. J.; Iyer, R.; Thomas, B. C.; Brown, C. T.; Anantharaman, K.; Tringe, S.; Hettich, R. L.; Harrison, S. T.; Banfield, J. F., Genome-resolved meta-omics ties microbial dynamics to process performance in biotechnology for thiocyanate degradation. *Environ Sci Technol* **2017,** *51* (5), 2944-2953.
26. Wang, Z.; Ho, H.; Egan, R.; Yao, S.; Kang, D.; Froula, J.; Sevim, V.; Schulz, F.; Shay, J. E.; Macklin, D.; McCue, K.; Orsini, R.; Barich, D. J.; Sedlacek, C. J.; Li, W.; Morgan-Kiss, R.; Woyke, T.; Slonczewski, J. L., A new method for rapid genome classification, clustering, visualization, and novel taxa discovery from metagenome. *biorXiv* **2019**.
27. Rossmassler, K.; Snow, C. D.; Taggart, D.; Brown, C.; De Long, S. K., Advancing biomarkers for anaerobic o-xylene biodegradation via metagenomic analysis of a methanogenic consortium. *Appl Microbiol Biotechnol* **2019,** *103* (10), 4177-4192.
28. Borton, M. A.; Hoyt, D. W.; Roux, S.; Daly, R. A.; Welch, S. A.; Nicora, C. D.; Purvine, S.; Eder, E. K.; Hanson, A. J.; Sheets, J. M.; Morgan, D. M.; Wolfe, R. A.; Sharma, S.; Carr, T. R.; Cole, D. R.; Mouser, P. J.; Lipton, M. S.; Wilkins, M. J.; Wrighton, K. C., Coupled laboratory and field investigations resolve microbial interactions that underpin persistence in hydraulically fractured shales. *Proc Natl Acad Sci U S A* **2018,** *115* (28), E6585-E6594.
29. Momper, L.; Jungbluth, S. P.; Lee, M. D.; Amend, J. P., Energy and carbon metabolisms in a deep terrestrial subsurface fluid microbial community. *ISME J* **2017,** *11* (10), 2319-2333.
30. FNR CORE 2011 project GASPOP (C11/SR/1280949: influence of the reactor design and the operational parameters on the dynamics of the microbial consortia involved in the biomethanation process).

31. BCO-DMO https://www.bco-dmo.org/dataset/716766.
32. Hawley, E. R.; Piao, H.; Scott, N. M.; Malfatti, S.; Pagani, I.; Huntemann, M.; Chen, A.; Glavina Del Rio, T.; Foster, B.; Copeland, A.; Jansson, J.; Pati, A.; Tringe, S.; Gilbert, J. A.; Lorenson, T. D.; Hess, M., Metagenomic analysis of microbial consortium from natural crude oil that seeps into the marine ecosystem offshore Southern California. *Stand Genomic Sci* **2014,** *9* (3), 1259-74.
33. Liu, T.; Ahn, H.; Sun, W.; McGuinness, L. R.; Kerkhof, L. J.; Haggblom, M. M., Identification of a ruminococcaceae species as the methyl tert-butyl ether (MTBE) degrading bacterium in a methanogenic consortium. *Environ Sci Technol* **2016,** *50* (3), 1455-64.
34. Dalcin Martins, P.; Danczak, R. E.; Roux, S.; Frank, J.; Borton, M. A.; Wolfe, R. A.; Burris, M. N.; Wilkins, M. J., Viral and metabolic controls on high rates of microbial sulfur and carbon cycling in wetland ecosystems. *Microbiome* **2018,** *6* (1), 138.
35. Fortney, N. W.; He, S.; Converse, B. J.; Boyd, E. S.; Roden, E. E., Investigating the composition and metabolic potential of microbial communities in Chocolate Pots Hot Springs. *Front Microbiol* **2018,** *9*, 2075.
36. Stern, N.; Mejia, J.; He, S.; Yang, Y.; Ginder-Vogel, M.; Roden, E. E., Dual role of humic substances as electron donor and shuttle for dissimilatory iron reduction. *Environ Sci Technol* **2018,** *52* (10), 5691-5699.
37. Frank, J. A.; Arntzen, M. O.; Sun, L.; Hagen, L. H.; McHardy, A. C.; Horn, S. J.; Eijsink, V. G.; Schnurer, A.; Pope, P. B., Novel syntrophic populations dominate an ammonia-tolerant methanogenic microbiome. *mSystems* **2016,** *1* (5).
38. Mackelprang, R.; Burkert, A.; Haw, M.; Mahendrarajah, T.; Conaway, C. H.; Douglas, T. A.; Waldrop, M. P., Microbial survival strategies in ancient permafrost: insights from metagenomics. *ISME J* **2017,** *11* (10), 2305-2318.
39. Reiss, R. A.; Guerra, P.; Makhnin, O., Metagenome phylogenetic profiling of microbial community evolution in a tetrachloroethene-contaminated aquifer responding to enhanced reductive dechlorination protocols. *Stand Genomic Sci* **2016,** *11*, 88.
40. Kleindienst, S.; Higgins, S. A.; Tsementzi, D.; Chen, G.; Konstantinidis, K. T.; Mack, E. E.; Loffler, F. E., '*Candidatus* Dichloromethanomonas elyunquensis' gen. nov., sp. nov., a dichloromethane-degrading anaerobe of the *Peptococcaceae* family. *Syst Appl Microbiol* **2017,** *40* (3), 150-159.
41. Liang, X.; Whitham, J. M.; Holwerda, E. K.; Shao, X.; Tian, L.; Wu, Y. W.; Lombard, V.; Henrissat, B.; Klingeman, D. M.; Yang, Z. K.; Podar, M.; Richard, T. L.; Elkins, J. G.; Brown, S. D.; Lynd, L. R., Development and characterization of stable anaerobic thermophilic methanogenic microbiomes fermenting switchgrass at decreasing residence times. *Biotechnol Biofuels* **2018,** *11*, 243.
42. Hawley, A. K.; Torres-Beltran, M.; Zaikova, E.; Walsh, D. A.; Mueller, A.; Scofield, M.; Kheirandish, S.; Payne, C.; Pakhomova, L.; Bhatia, M.; Shevchuk, O.; Gies, E. A.; Fairley, D.; Malfatti, S. A.; Norbeck, A. D.; Brewer, H. M.; Pasa-Tolic, L.; Del Rio, T. G.; Suttle, C. A.; Tringe, S.; Hallam, S. J., A compendium of multi-omic sequence information from the Saanich Inlet water column. *Sci Data* **2017,** *4*, 170160.
43. He, S.; Malfatti, S. A.; McFarland, J. W.; Anderson, F. E.; Pati, A.; Huntemann, M.; Tremblay, J.; Glavina del Rio, T.; Waldrop, M. P.; Windham-Myers, L.; Tringe, S. G., Patterns in wetland microbial community composition and functional gene repertoire associated with methane emissions. *MBio* **2015,** *6* (3), e00066-15.

44. Nobu, M. K.; Narihiro, T.; Rinke, C.; Kamagata, Y.; Tringe, S. G.; Woyke, T.; Liu, W. T., Microbial dark matter ecogenomics reveals complex synergistic networks in a methanogenic bioreactor. *ISME J* **2015,** *9* (8), 1710-22.
45. Lykidis, A.; Chen, C. L.; Tringe, S. G.; McHardy, A. C.; Copeland, A.; Kyrpides, N. C.; Hugenholtz, P.; Macarie, H.; Olmos, A.; Monroy, O.; Liu, W. T., Multiple syntrophic interactions in a terephthalate-degrading methanogenic consortium. *ISME J* **2011,** *5* (1), 122-30.
46. DeAngelis, K. M.; Fortney, J. L.; Borglin, S.; Silver, W. L.; Simmons, B. A.; Hazen, T. C., Anaerobic decomposition of switchgrass by tropical soil-derived feedstock-adapted consortia. *MBio* **2012,** *3* (1).
47. Deangelis, K. M.; D'Haeseleer, P.; Chivian, D.; Fortney, J. L.; Khudyakov, J.; Simmons, B.; Woo, H.; Arkin, A. P.; Davenport, K. W.; Goodwin, L.; Chen, A.; Ivanova, N.; Kyrpides, N. C.; Mavromatis, K.; Woyke, T.; Hazen, T. C., Complete genome sequence of "Enterobacter lignolyticus" SCF1. *Stand Genomic Sci* **2011,** *5* (1), 69-85.
48. DeAngelis, K. M.; Allgaier, M.; Chavarria, Y.; Fortney, J. L.; Hugenholtz, P.; Simmons, B.; Sublette, K.; Silver, W. L.; Hazen, T. C., Characterization of trapped lignin-degrading microbes in tropical forest soil. *PLoS One* **2011,** *6* (4), e19306.
49. DeAngelis, K. M.; Gladden, J. M.; Allgaier, M.; D'haeseleer, P.; Fortney, J. L.; Reddy, A.; Hugenholtz, P.; Singer, S. W.; Vander Gheynst, J. S.; Silver, W. L.; Simmons, B. A.; Hazen, T. C., Strategies for Enhancing the Effectiveness of Metagenomic-based Enzyme Discovery in Lignocellulolytic Microbial Communities. *BioEnergy Research* **2010,** *3* (2), 146-158.
50. Spang, A.; Saw, J. H.; Jorgensen, S. L.; Zaremba-Niedzwiedzka, K.; Martijn, J.; Lind, A. E.; van Eijk, R.; Schleper, C.; Guy, L.; Ettema, T. J. G., Complex archaea that bridge the gap between prokaryotes and eukaryotes. *Nature* **2015,** *521* (7551), 173-179.
51. Barnum, T. P.; Figueroa, I. A.; Carlstrom, C. I.; Lucas, L. N.; Engelbrektson, A. L.; Coates, J. D., Genome-resolved metagenomics identifies genetic mobility, metabolic interactions, and unexpected diversity in perchlorate-reducing communities. *ISME J* **2018,** *12* (6), 1568-1581.
52. Levy-Booth, D. J.; Giesbrecht, I. J. W.; Kellogg, C. T. E.; Heger, T. J.; D'Amore, D. V.; Keeling, P. J.; Hallam, S. J.; Mohn, W. W., Seasonal and ecohydrological regulation of active microbial populations involved in DOC, CO_2, and CH_4 fluxes in temperate rainforest soil. *ISME J* **2019,** *13* (4), 950-963.
