# Supplementary material for: Structure determination of the HgcAB complex using metagenome sequence data: insights into microbial mercury methylation
Source: Commun Biol. 2020 Jun 19;3:320. doi: 10.1038/s42003-020-1047-5 (PMC7305189; doi:10.1038/s42003-020-1047-5)
Supplement: Supplementary file 2 — Description of Additional Supplementary Files [file 42003_2020_1047_MOESM2_ESM.pdf]

Description of Additional Supplementary Files

File Name: Supplementary Data 1
Description: Data used to generate plots in Figures 1 and 2

File Name: Supplementary Data 2
Description: HgcAB multiple sequence alignment in fasta format

File Name: Supplementary Data 3
Description: HgcA-only multiple sequence alignment in fasta format

File Name: Supplementary Data 4
Description: Complete list of metagenomic datasets and associated references
