# Supplementary material for: Structure determination of the HgcAB complex using metagenome sequence data: insights into microbial mercury methylation
Source: Commun Biol. 2020 Jun 19;3:320. doi: 10.1038/s42003-020-1047-5 (PMC7305189; doi:10.1038/s42003-020-1047-5)
Supplement: Supplementary file 8 — Reporting Summary [file 42003_2020_1047_MOESM8_ESM.pdf]

nature research

Corresponding author(s): Jerry M. Parks

Last updated by author(s): Apr 27, 2020

Reporting Summary

Nature Research wishes to improve the reproducibility of the work that we publish. This form provides structure for consistency and transparency in reporting. For further information on Nature Research policies, see Authors & Referees and the Editorial Policy Checklist .

Statistics

For all statistical analyses, confirm that the following items are present in the figure legend, table legend, main text, or Methods section.

n/a	Confirmed	
☒	☐	The exact sample size (*n*) for each experimental group/condition, given as a discrete number and unit of measurement
☒	☐	A statement on whether measurements were taken from distinct samples or whether the same sample was measured repeatedly
☒	☐	The statistical test(s) used AND whether they are one- or two-sided<br>*Only common tests should be described solely by name; describe more complex techniques in the Methods section.*
☒	☐	A description of all covariates tested
☒	☐	A description of any assumptions or corrections, such as tests of normality and adjustment for multiple comparisons
☒	☐	A full description of the statistical parameters including central tendency (e.g. means) or other basic estimates (e.g. regression coefficient) AND variation (e.g. standard deviation) or associated estimates of uncertainty (e.g. confidence intervals)
☒	☐	For null hypothesis testing, the test statistic (e.g. *F*, *t*, *r*) with confidence intervals, effect sizes, degrees of freedom and *P* value noted<br>*Give P values as exact values whenever suitable.*
☒	☐	For Bayesian analysis, information on the choice of priors and Markov chain Monte Carlo settings
☒	☐	For hierarchical and complex designs, identification of the appropriate level for tests and full reporting of outcomes
☒	☐	Estimates of effect sizes (e.g. Cohen's *d*, Pearson's *r*), indicating how they were calculated

Our web collection on statistics for biologists contains articles on many of the points above.

Software and code

Policy information about availability of computer code

Data collection	We used hhblits and hhfilter from HH-suite (https://github.com/soedinglab/hh-suite), hmmbuild and hmmsearch from HMMER version 3.1 (http://hmmer.org/download.html), GREMLIN 2.01 (available at http://gremlin.bakerlab.org/gremlin.php), map_align (available at https://github.com/sokrypton/map_align), and Rosetta 3.8.
Data analysis	TM-score (available at https://zhanglab.ccmb.med.umich.edu/TM-score/), Muscle v. 3.8.425 (https://www.drive5.com/muscle/downloads.htm), Geneious (https://www.geneious.com), FastTree v. 2.1.12 (http://www.microbesonline.org/fasttree/), and iTOL (https://itol.embl.de)

For manuscripts utilizing custom algorithms or software that are central to the research but not yet described in published literature, software must be made available to editors/reviewers. We strongly encourage code deposition in a community repository (e.g. GitHub). See the Nature Research guidelines for submitting code & software for further information.

Data

Policy information about availability of data

All manuscripts must include a data availability statement. This statement should provide the following information, where applicable:

- Accession codes, unique identifiers, or web links for publicly available datasets
- A list of figures that have associated raw data
- A description of any restrictions on data availability

The paired HgcAB multiple sequence alignment data, HgcAB structural model, HgcA-only multiple sequence alignment, and a complete list of metagenome datasets and associated references are provided as supplementary files.

Field-specific reporting

Please select the one below that is the best fit for your research. If you are not sure, read the appropriate sections before making your selection.

☐ Life sciences ☐ Behavioural & social sciences ☒ Ecological, evolutionary & environmental sciences

For a reference copy of the document with all sections, see nature.com/documents/nr-reporting-summary-flat.pdf

Ecological, evolutionary & environmental sciences study design

All studies must disclose on these points even when the disclosure is negative.

Study description	We searched metagenomes for sequences of HgcA and HgcB. Using residue-residue contacts inferred from coevolution analysis of the resulting sequence alignment, we generated a 3D model of the complex. In addition, we expressed these two proteins in E. coli and confirmed cofactor binding spectroscopically.
Research sample	A master database consisting of the Uniref100 database and the JGI Metagenome database was searched computationally to identify sequence homologs of HgcA and HgcB.
Sampling strategy	To remove highly similar sequences, which provide no benefit for coevolution analysis, we used the program hhfilter with a sequence identity cutoff of 90%. The resulting multiple sequence alignment is provided as supporting information
Data collection	We used publicly available metagenome data from the U.S. Department of Energy (DOE) Joint Genome Institute (JGI).
Timing and spatial scale	The metagenome sequence searches were performed in January 2018.
Data exclusions	Initial searches identified 7,505 and 19,317 putative HgcA and HgcB sequences, respectively. We then exploited co-occurrence and adjacency to generate a paired alignment of HgcA and HgcB. After pairing of HgcA and HgcB sequences based on whether two hits were from the same metagenomic contig, we obtained 3,025 sequences. We used 90% identity filtering to remove redundant sequences (2,432)
Reproducibility	The multiple sequence alignment of paired HgcA and HgcB sequences is provided as supporting information. The coevolution analysis can be reproduced readily using the program GREMLIN. Models can be generated by following the protocol described in the paper and in references provided.
Randomization	Due to the nature of this work, no randomization was required.
Blinding	Due to the nature of this work, no blinding was required.

Did the study involve field work? ☐ Yes ☒ No

Reporting for specific materials, systems and methods

We require information from authors about some types of materials, experimental systems and methods used in many studies. Here, indicate whether each material, system or method listed is relevant to your study. If you are not sure if a list item applies to your research, read the appropriate section before selecting a response.

Materials & experimental systems

n/a	Involved in the study
☐	☒ Antibodies
☒	☐ Eukaryotic cell lines
☒	☐ Palaeontology
☒	☐ Animals and other organisms
☒	☐ Human research participants
☒	☐ Clinical data

Methods

n/a	Involved in the study
☒	☐ ChIP-seq
☒	☐ Flow cytometry
☒	☐ MRI-based neuroimaging

Antibodies

Antibodies used	An antibody against the His-tag in heterologously expressed proteins was used according to the manufacturer's instructions for western blot analyses.
Validation	We used a standard, commercially available monoclonal anti-polyhistidine-peroxidase conjugate antibody from Sigma-Aldrich (catalog number A7058-1VL, lot number 077M4847V). Information is provided here: https://www.sigmaaldrich.com/content/dam/sigma-aldrich/docs/Sigma/Datasheet/6/a7058dat.pdf
